# Microstructure and Properties of Hot Pressing Sintered SiC/Y_3_Al_5_O_12_ Composite Ceramics for Dry Gas Seals

**DOI:** 10.3390/ma17051182

**Published:** 2024-03-03

**Authors:** Chang Zou, Yangxin Ou, Weiliang Zhou, Zhiqiang Li, Pu Zheng, Xingzhong Guo

**Affiliations:** 1 School of Materials Science and Engineering, Zhejiang University, Hangzhou 310058, China; 0924054@zju.edu.cn (C.Z.); 22226070@zju.edu.cn (Y.O.); 2 ZJU-Hangzhou Global Scientific and Technological Innovation Center, Hangzhou 311200, China; 3 Zhejiang Dongxin New Material Technology Co., Ltd., Taizhou 318000, China; dongxin58@dongxin.com (W.Z.); dongxin27@dongxin.com (Z.L.); dongxin1@dongxin.com (P.Z.)

**Keywords:** silicon carbide, hot pressing sintering, yttrium aluminum garnet, ceramic material, microstructure

## Abstract

Silicon carbide (SiC) ceramics with high bending strength were prepared by hot pressing sintering (HPS) with yttrium aluminum garnet (Y_3_Al_5_O_12_, YAG) as sintering additive, and the effects of YAG content and sintering temperature on the sintering behavior, microstructure and mechanical properties of SiC ceramics were investigated in detail. The uniform distribution of YAG to form a liquid phase and the driving force provided by hot pressing sintering decrease the sintering temperature, improve the densification of SiC ceramics, and refine the crystal size. By means of suitable sintering conditions with the additional amount of YAG of 5 wt%, the sintering temperature of 1950 °C and a pressure of 30 MPa, the resultant SiC/YAG composite ceramics possesses high sintering and mechanical properties with the relative density of 98.53%, the bending strength of 675 MPa, the Vickers hardness of up to 17.92 GPa, and the elastic modulus of 386 GPa. The as-prepared SiC/YAG composite ceramics are promisingly used as the dry gas seal materials in the centrifugal compressors.

## 1. Introduction

The dry gas seal is a non-contact seal that improves the mechanical seal based on gas dynamic pressure bearing. It exhibits high ultimate speed, excellent sealing performance, and a long lifespan. It eliminates the need for an oil sealing system, consumes less power, and offers simple operation and low maintenance costs. Consequently, it has gained extensive utilization in the petroleum industry. The sealing materials for the dynamic and static rings of dry gas seals need to have sufficient strength and elastic modulus [1]. It ensures that the sealing materials can not be damaged under certain pressure, temperature, and high-speed rotation conditions and have minimal deformation and the ability to maintain sealing even under fluctuating operating conditions [2,3,4]. Currently, the static and dynamic rings of dry gas seals are primarily made of advanced structural ceramic materials. Due to high rotational speed, dry gas seals have strict requirements for the mechanical properties of ceramic sealing materials, especially bending strength. High-strength sealing materials have become one of the main development directions for dry gas seals [5,6,7].

Silicon carbide (SiC) ceramic is an excellent structural material with excellent high-temperature mechanical properties, high thermal conductivity, good thermal shock resistance, chemical corrosion resistance, low thermal expansion coefficient, and light-specific gravity, and becomes one of the basic sealing materials [2,3,8]. However, low room temperature strength, low fracture toughness, and lack of plastic deformation ability seriously restrict the application of silicon carbide ceramic as a dry gas sealing material.

As is well known, silicon carbide ceramics are difficult to densify without any special treatment due to the high covalent bond of Si-C. Thus, how to obtain a greater sintering driving force during the sintering process is the most important thing to obtain high-density silicon carbide ceramics [2,8,9,10]. Among the studies on improving the sintering driving force, the research of appropriate sintering methods and sintering additives have become hot spots in this field. The main sintering methods of SiC ceramics are pressureless sintering [11,12,13] and reaction sintering [14,15], but the two sintering have low purity. To avoid these drawbacks, researchers have developed the hot pressing sintering method [9,16,17], spark plasma sintering [18,19,20], microwave sintering [21], hot isostatic sintering [22], and so on. Each of these techniques offers its own set of advantages and disadvantages, with numerous valuable research findings to support their efficacy. Nevertheless, the underlying principle driving these techniques is similar, which is to enhance the sintering driving force and improve the density of the final ceramic material.

Among advanced sintering technologies, hot pressing sintering is a mechanical pressure sintering method, which puts the ceramic powder or green body in the mold cavity with pressure assistance [23,24,25,26]. Due to the driving force supplied by the external pressure, the compaction can be achieved in a short time, and the microstructure with fine and uniform crystals can be obtained [27,28,29,30]. This sintering method can obtain better mechanical properties of materials, reduce sintering time and temperature, decrease the amount of sintering additives, and improve the high-temperature mechanical properties of materials [31,32,33]. Among the sintering additives of silicon carbide ceramics, yttria/alumina oxides have been widely used in pressureless liquid sintering, which are added to decrease the sintering temperature and improve the fracture toughness of silicon carbide ceramics due to the formation of an in situ YAG phase [34,35]. At a lower temperature (1760 °C), the added Al_2_O_3_ and Y_2_O_3_ can react to form aluminum yttrium garnet (YAG, Y_3_Al_5_O_12_), and the YAG exists in the form of a liquid phase at the sintering temperature of silicon carbide ceramics. This phenomenon plays a crucial role in promoting mass transfer during the sintering process, which significantly contributes to the densification of the silicon carbide ceramic materials. As a result, the mechanical properties of the ceramic materials are improved [36,37]. However, there are few reports on the application of YAG as a sintering additive in the hot pressing sintering of silicon carbide ceramics for dry gas seals.

In this work, we demonstrate the preparation of SiC/YAG composite ceramics by hot pressing sintering. The yttrium/alumina oxides are uniformly dispersed in SiC powder by ball milling and spray drying, and the mixed powder is sintered into high-density SiC ceramic materials by hot pressing sintering. The effects of sintering temperature and the YAG content on the sintering behavior, microstructure, and properties of SiC/YAG composite ceramics are investigated at great length.

## 2. Materials and Methods

### 2.1. Preparation of Silicon Carbide Composite Ceramic Materials

α-SiC powder (with an average particle size of 0.17 μm) was provided by Zhejiang Dongxin New Material Technology Co., Ltd. (Linhai, China), Al_2_O_3_ and Y_2_O_3_ were purchased from the Sinopharm (Shanghai, China), and polyethylene oxide (PEO) and polyvinyl alcohol (PVA) were purchased from the Aladin (Shanghai, China). In the experiment, 87 wt% and 92 wt% α-SiC powder, 5 wt% and 10 wt% Al_2_O_3_ and Y_2_O_3_ mixed powder, 1 wt% PEO, and 2 wt% PVA were mixed in a certain proportion according to the stoichiometry of 5 wt% and 10 wt% YAG (the mole ratio of Y:Al is 1:1), respectively. After mixing for 4 h by ball milling with deionized water, a slurry with 40% solid phase content was obtained. The obtained aqueous slurry was spray-dried to prepare the mixed granulation powder. The mixed powder was uniaxially pressed at 100 MPa in a carbon steel die into rectangular specimens, and then these green bodies were compacted at 250 MPa in a cold isostatic press. The green body was sintered in the hot pressing sintering furnace with Ar condition, which was heated up to 400 °C with a heating rate of 6 °C/min. After insulation for 15 min, the furnace was heated up to the sintering temperature (1900, 1950, or 2000 °C) at 10 °C/min, and the sintering time was 1 h. In addition, before being heated, the sample was pre-loaded with a pressure of 30 MPa for 5 min. After that, an initial external pressure of 10 MPa was applied at the beginning of the heating, which was continuously increased as the heating process proceeded, and the external pressure was 30 MPa at the holding stage and maintained until the end of the sintering process. After cooling, demolding, and processing, silicon carbide composite ceramic materials were obtained.

### 2.2. Characterization

The conventional water displacement method was used to measure the volume density of the green body and sintered body to obtain the relative density of the ceramic material. Phase identification was performed by the X-ray diffraction (XRD) method on a Rigaku D/max-RA X-ray diffractometer (Rigaku, Japan) using nickel-filtered Cu-Kα radiation in the range of 10~80° at a scanning rate of 2° per minute. The morphology and distribution of elements of the sintered samples were observed by scanning electron microscopy (SEM, Hitachi S-4800, Hitachi, Japan). Bending strength and elastic modulus were measured by three-point bending tests with a 30 mm span at a cross-head speed of 0.5 mm/min using CMT5205 electronic universal testing machine with 10 samples in each group were tested, and the average value was taken. The microhardness of SiC ceramic materials was measured by an HV-5 Vickers hardness tester with a load of 98 N and a loading time of 15 s with 5 samples in each group tested, and the average value was taken. The fracture toughness of the material was calculated by the following formula [38]:

(1) $$K_{IC}=\frac{0.129}{3}{\left(\frac{c}{a}\right)}^{-\frac{3}{2}}H\sqrt{a}{\left(\frac{H}{3E}\right)}^{-0.4}\;\;\;\;\;(\frac{c}{a}>2.5)$$

(2) $$K_{IC}=\frac{0.035}{3}{\left(\frac{l}{a}\right)}^{-\frac{1}{2}}H\sqrt{a}{\left(\frac{H}{3E}\right)}^{-0.4}\;\;\;\;\;(\frac{c}{a}<2.5)$$

In the formula, *K*_IC_ is the fracture toughness of the testing material, *H* is the hardness of the testing material, *E* is the elastic modulus of the testing material, *l* is the indentation crack length, *a* is the half-length of the indentation diagonal, and *c* = 1 + *a*.

## 3. Results and Discussion

### 3.1. Preparation of SiC/YAG Composite Ceramics by Hot Pressing Sintering

**Figure 1:**
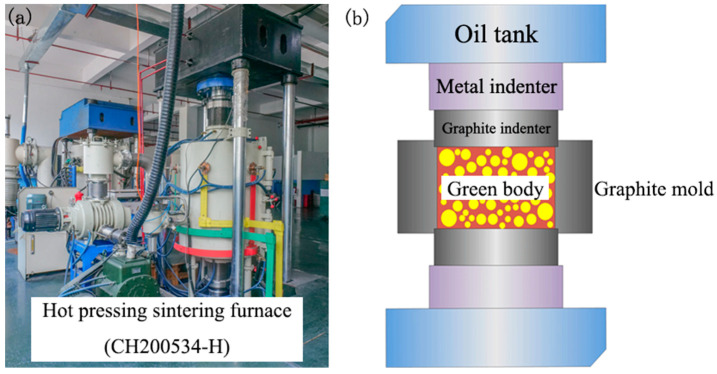
Photo (**a**) and structure diagram (**b**) of hot pressing sintering furnace.

The hot pressing sintering furnace and graphite mold used in this work are shown in Figure 1. To facilitate demolding after sintering, a layer of graphite paper is placed around the graphite mold, and another layer is laid at the bottom of the mold. The green body is then placed inside, followed by additional layers of graphite paper, a graphite pad, and a graphite indenter in sequence. The graphite mold with the green body is put into the hot pressing furnace, and the vacuum pressure is applied to create negative pressure. Before starting the sintering process, an external pressure of 30 MPa is applied for pre-loading for 5 min. After that, the temperature is raised to 400 °C at a heating rate of 6 °C/min and maintained for 15 min to eliminate organic binder and dispersant from the green body, which improves density and purity for subsequent ceramic sintering. Then, the temperature of the hot pressing sintering furnace is increased to reach the sintering temperature at a rate of 10 °C/min and maintained for one hour. As a result, the SiC/YAG composite ceramics can be achieved. Herein, the effect of the content of YAG and the sintering temperature on the sintering behavior, microstructure, and mechanical properties of SiC/YAG composite ceramics were investigated in detail.

In this work, a mixed powder composed of Al_2_O_3_ and Y_2_O_3_ was mixed with silicon carbide powder as a sintering additive. In the sintering process, Al_2_O_3_ and Y_2_O_3_ will react at a high temperature to form YAG in a liquid phase at a high temperature. The liquid phase component can accelerate the mass transfer of crystals and promote the combination of crystals. On the one hand, the crystals are not easy to grow and are small and uniform. At the same time, the introduction of the liquid phase in the grain boundary and the unique interface structure lead to the weakening of the interface bond, and the fracture of the material becomes a complete intergranular fracture mode, resulting in a significant increase in the strength and toughness of the material [17].

As one of the specialized sintering methods distinct from conventional sintering, hot pressing sintering involves simultaneous heating of ceramic powder and application of pressure. Under the combined influence of temperature and pressure, the powder particles in the graphite grinding tool gradually approach each other, undergo slip and deformation, and rely on various mass transfer mechanisms to accomplish densification. Consequently, hot pressing sintering reduces diffusion distance, accelerates plastic flow processes, and promotes the acceleration of other mass transfer processes. The sintering temperature of hot pressing sintering is typically 150–200 °C lower than that of conventional sintering, and the sintering time is significantly shorter than conventional sintering. In comparison to conventional sintered bodies, hot pressing sintered bodies exhibit low porosity, high relative density, reduced sintering temperatures, shorter sintering times, and limited grain growth potential, resulting in superior mechanical properties.

### 3.2. Sintering Behavior of Hot Pressing Sintered SiC/YAG Composite Ceramics

**Figure 2:**
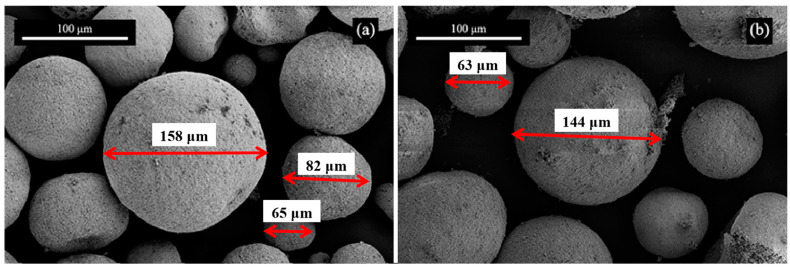
SEM photos of SiC/YAG composite powder. (**a**) 5 wt% YAG; (**b**) 10 wt% YAG.

**Figure 3:**
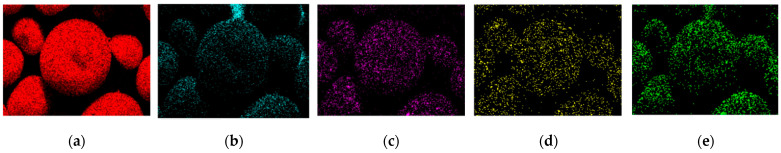
EDS of SiC/YAG composite powder (5 wt% YAG). (**a**) Si; (**b**) C; (**c**) Al; (**d**) Y; (**e**) O.

The morphology of SiC/YAG composite powders prepared by ball milling and spray drying is shown in Figure 2. The particle size of granulated composite powder is between 50 and 150 μm, and the shape is regular spherical, which indicates good fluidity and high fillability. According to the sintering mechanism, the quality of sintered ceramic products is related to the particle size of raw materials, sintering additives, dispersion procedure, and sintering procedure [33]. The effect of sintering additives on ceramic sintering comes from its content and distribution uniformity. Thus, the distribution of additives with the same content is very important for sintering. In this study, after the procedure of ball milling and spray drying, the sintering additives can be uniformly distributed in the composite powder, which ensures the formation and uniformity of the YAG liquid phase during sintering and improves the sintering performance of SiC ceramics. From the EDS analysis (Figure 3), the distribution of yttrium, aluminum, and SiC in the composite powder is very uniform. The uniform distribution of sintering additives in SiC powder can effectively reduce the sintering temperature and time and improve the sintering performance of SiC composites.

The sintering properties and crystalline phases of the SiC ceramic materials are given in Table 1. The SiC ceramic sample with 5 wt% YAG and sintered at 1950 °C possesses the highest density (3.223 g/cm^3^). From Table 1, the ceramic samples obtained at 1950 °C are significantly denser than the ceramic samples obtained at 1900 and 2000 °C. The weight loss ratio presented in Table 1 reflects the extent of mass reduction experienced by the sintered ceramic as compared to the ceramic green body before and after the sintering process. The weight loss ratio of the samples sintered at 1900 and 1950 °C is relatively close, while the sample sintered at 2000 °C has a higher weight loss rate, which results from the volatilization of sintering additive at high temperatures. During the sintering process, the sintering temperature can significantly affect the sintering properties and microstructure of SiC ceramics. At high temperatures, the densification of YAG is reinforced, but it may result in the overgrowth of crystals, the volatilization of the YAG phase, and the reaction of SiC with other oxides, which may cause defects to worsen the properties of ceramics. However, at low temperatures, the content of the YAG liquid phase and the sintering driving force caused by temperature are insufficient, which obstructs the densification of SiC ceramic. In comparison, the sintering temperature of 1950 °C can meet the requirement of densification, and it is the suitable sintering temperature for SiC/YAG composite ceramic.

**Figure 4:**
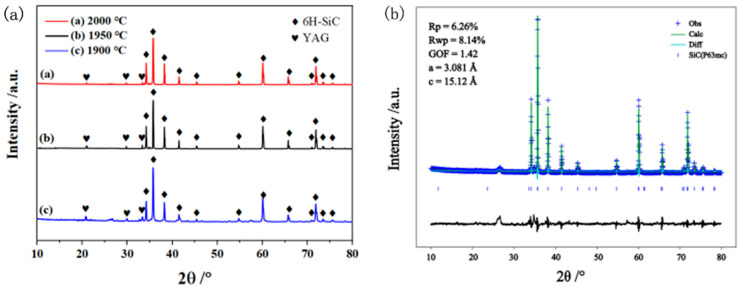
XRD patterns of SiC/YAG composite ceramics (5 wt% YAG) after hot pressing sintered at different temperatures (**a**) and refined XRD data of SiC/YAG composite ceramics sintered at 1950 °C (**b**).

From the XRD diffraction patterns in Figure 4 and Table 1, it is evident that the primary phase of SiC/YAG composite ceramics is 6H-SiC, while the secondary phase consists of YAG crystal formed by Al_2_O_3_ and Y_2_O_3_ as sintering additives. Based on the refined XRD data obtained from the ceramic sample with a holding temperature of 1950 °C, the lattice constants of the SiC crystal are found to be *a* = 3.081 Å and *c* = 12.12 Å, belonging to the P63mc space group. It also can be seen from Table 1 that the increase in sintering temperature obviously causes more weight loss, but the bulk density and relative density are not improved, as mentioned before. Additionally, Figure 4 demonstrates a gradual decrease in peak intensity for each characteristic peak of YAG with increasing sintering temperature, confirming that at higher temperatures, YAG generated from Al_2_O_3_ and Y_2_O_3_ reacts and volatilizes to some extent, causing a certain degree of loss. The significant loss of numerous sintering additives during the dense sintering process for silicon carbide ceramics is evidently unfavorable; however, on the other hand, reducing the presence of weak mechanical properties associated with YAG crystal phases also contributes to improving the mechanical properties of silicon carbide ceramics to some extent. Therefore, further exploration through testing is required to determine the actual impact this issue has on performance.

### 3.3. Microstructure of Hot Pressing Sintered SiC/YAG Composite Ceramic

In the hot pressing sintering process, the SiC/YAG powder is in a thermoplastic state and is endowed with low deformation resistance and easy plastic flow and densification. High temperature and pressurization facilitate mass transfer processes such as contact, diffusion, and flow of powder particles, reducing sintering temperature and shortening sintering time. The densification inhibits grain growth. Therefore, the sintering temperature and the proportion of the sintering additives have a significant effect on the microstructure of the SiC ceramic materials.

**Figure 5:**
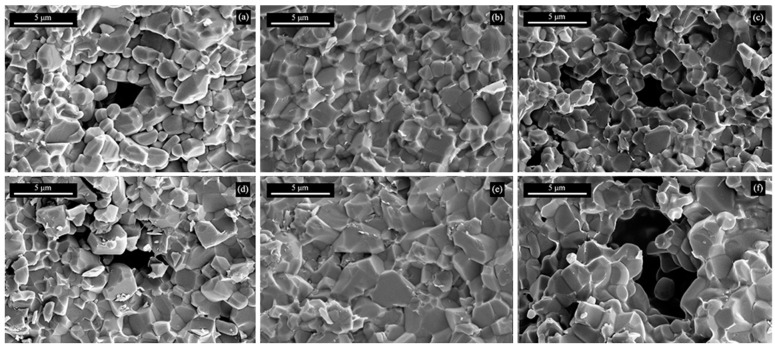
SEM photos of the fracture surface of SiC/YAG composite ceramics after hot pressing sintered at different YAG contents and sintering temperatures. (**a**) 5 wt% YAG, 1900 °C; (**b**) 5 wt% YAG, 1950 °C; (**c**) 5 wt% YAG, 2000 °C; (**d**) 10 wt% YAG, 1900 °C; (**e**) 10 wt% YAG, 1950 °C; (**f**) 10 wt% YAG, 2000 °C.

Figure 5 shows SEM photos of the fracture surface of the SiC/YAG composite ceramics sintered at different temperatures. The crystal size of SiC/YAG composite ceramics mainly distributes in 2~3 μm. At the same sintering temperature, the SiC ceramic samples with 10 wt% YAG added have relatively larger crystal sizes compared with SiC ceramic samples with 5 wt% YAG. This is because the larger proportion of YAG can promote mass transfer and crystal growth in the sintering process. At the same YAG content, with the increase of the sintering temperature, the sintering driving force of the ceramic is stronger, so the ceramic at high temperature has a larger crystal size, and the combination between crystals becomes more compact. It can also be seen that there are obvious pores in the SiC ceramic samples sintered at 1900 and 2000 °C. When the temperature is 1900 °C, the green body is unable to achieve sufficient sintering densification with large pores and loose crystal distribution, which indicates that the matrix crystals can not be tightly bonded. When the sintering temperature is too high, more YAG becomes the liquid phase to improve the sintering of silicon carbide ceramic, while some YAG will volatilize to form large pores, and the overgrowth of crystal occurs. At the sintering temperature of 1950 °C, it can be observed that there is a small internal grain size and a fine crystal structure, indicating that the liquid phase content at this temperature can meet the requirements of ceramic to densify and the growth of crystal. Therefore, 1950 °C is a suitable sintering temperature for SiC/YAG composite powder.

### 3.4. Mechanical Properties of Hot Pressing Sintered SiC/YAG Composite Ceramic

**Figure 6:**
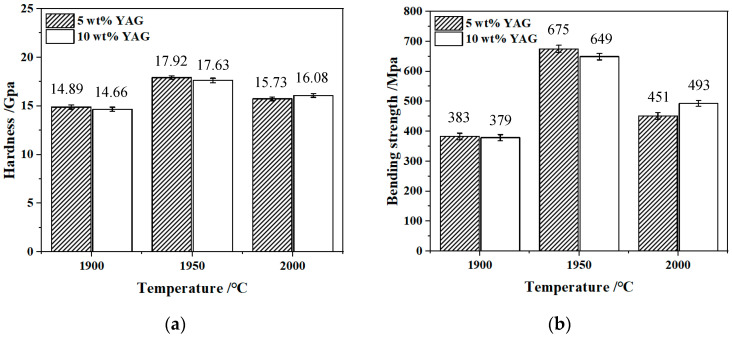
Hardness (**a**) and bending strength (**b**) of SiC/YAG composite ceramics after hot pressing sintered at different YAG contents and sintering temperatures.

Figure 6 displays the hardness and bending strength of SiC/YAG composite ceramics after hot pressing sintered at different YAG contents and sintering temperatures. It can be noted that the bending strength of the SiC ceramic sample firstly increases and then decreases with the increase of sintering temperature. For the sample with 5 wt% YAG added, the hardness of ceramic samples sintered at 1900, 1950, and 2000 °C is 14.89, 17.92, and 15.73 GPa, respectively. For the sample with 10 wt% YAG added, the hardness of ceramic samples sintered at 1900, 1950, and 2000 °C is 14.66, 17.63, and 16.08 GPa. At 1950 °C, the hardness of the SiC ceramics reaches the highest, indicating that the SiC ceramics sintered at this temperature are the densest and the surface defects are the least. In terms of bending strength, the bending strength of ceramics with 5 wt% and 10 wt% YAG added sintered at 1900, 1950, and 2000 °C is 383, 675, and 451 MPa, and 379, 649, and 493 MPa, respectively. At the same sintering temperature, the strength of the sample with 5 wt% YAG is higher than that of the sample with 10 wt% YAG. Due to a large number of grain boundaries and few internal defects, which can effectively absorb stress and curb the expansion of cracks, the performance in hardness and bending strength of the sample with 5 wt% YAG is stronger than other samples with larger grain size. Owing to the low density and the large number of internal pores between the crystal boundaries in the samples sintered at 1900 and 2000 °C, those two samples show low bending strength and hardness. It is worth noting that when the temperature rises to 2000 °C, the 10 wt% YAG sample has stronger mechanical properties, which may result from the stronger effect of the sintering additive on promoting the density of the ceramic at higher temperatures.

**Figure 7:**
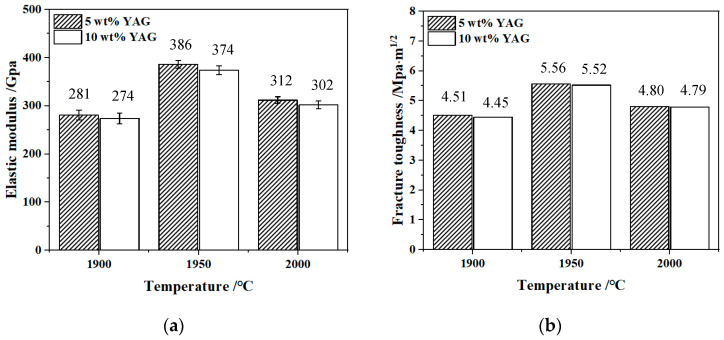
Elastic modulus (**a**) and fracture toughness (**b**) of SiC/YAG composite ceramics after hot pressing sintered at different YAG contents and sintering temperatures.

**Figure 8:**
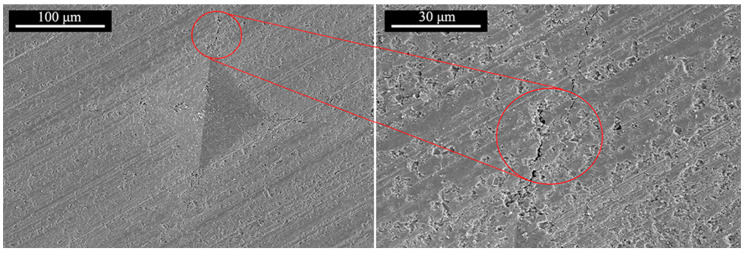
Crack deflecting of SiC/YAG composite ceramic (5 wt% YAG) after hot pressing sintered at 1950 °C.

Figure 7 shows the elastic modulus and fracture toughness of SiC/YAG composite ceramics after hot pressing sintered at different YAG contents and sintering temperatures. It is seen that the elastic modulus and fracture toughness show a trend of first increasing and then decreasing, with the increase of sintering temperature. When the sintering temperature is 1950 °C, the SiC ceramics with 5 wt% YAG have the highest elastic modulus and fracture toughness, which are 386 GPa and 5.56 MPa·m^1/2^, respectively. Numerous studies have shown that the strength of ceramics increases with decreasing crystal size and porosity [39]. The samples sintered at 1950 °C with 5 wt% YAG had a fine microstructure with a compact internal structure, fine grain size, and few pores, which improved bending strength and fracture toughness. According to the fracture mechanism, the finer grain and larger crystal boundary proportion will extend the crack propagation path. Furthermore, the cracks tend to break along grain boundaries, which causes the deflection of the main crack along the crystals. The deflection of the main crack will prolong its propagation path, as shown in Figure 8, thereby improving the fracture toughness of the material.

The mechanical properties of various SiC ceramics obtained by pressure-free and hot press sintering are presented in Table 2. Guo et al. prepared SiC ceramics through pressure-free sintering [40], uniformly dispersing YAG and silicon carbide powders using the sol–gel method. Despite the uniform dispersion of the YAG liquid phase within the SiC crystal, the resulting ceramic strength did not surpass that achieved in this study. Furthermore, the strength and toughness of SiC composite ceramics containing a nano-tin phase obtained through liquid phase sintering were also lower than those of pure SiC ceramics produced via hot pressing sintering [41]. The former indicates that although the mixed powder obtained through the sol–gel method is more uniform compared to simple ball milling, the driving force for sintering provided by pressure-free sintering is significantly less than that offered by hot pressing sintering. Consequently, the transmission speed of components within the green body during the sintering process is noticeably slower and insufficient, leading to inferior mechanical properties. The latter demonstrates that while adding reinforcing phases to compound SIC ceramics effectively enhances material strength, it remains notably lower than what can be achieved with hot pressing technology. Overall, performance comparison in Table 2 reveals that compared to mere improvements in formulation and dispersion processes, advancements in sintering technology can significantly enhance ceramic material strength, thus emphasizing how additional external pressure during the sintering process plays a crucial role in improving compaction and mechanical properties of ceramics. Moreover, combining hot pressing technology with the sol–gel method and composite ceramic technology holds the potential for obtaining even higher-performance silicon carbide composite ceramics.

## 4. Conclusions

In summary, hot pressing sintered SiC/YAG composite ceramics with outstanding mechanical properties had been successfully prepared with YAG as a sintering additive. The YAG content and sintering temperature have an important role in the sintering densification of SiC ceramics. When the sintering temperature is 1950 °C, the addition of YAG is 5 wt%, and the external pressure is 30 MPa, the resultant hot pressing sintered SiC/YAG composite ceramics have high sintering and mechanical properties. The bending strength of obtained SiC/YAG composite ceramics reaches 675 MPa, the surface Vickers hardness is 17.92 GPa, and the fracture toughness is 5.56 MPa·m^1/2^. The formed liquid phase YAG and additional high pressure promote quick densification of silicon carbide ceramic materials and achieve the refinement of internal crystals. The as-prepared SiC/YAG composite ceramics can be used as the sealing materials for dry gas seals.

## Figures and Tables

**Table 1:** Sintering properties and crystalline phases of SiC/YAG composite ceramic.

Sample		Bulk Density (g/cm^3^)	Weight-Loss Ratio (%)	Relative Density (%)	Crystalline Phases	
					Major	Secondary
SiC/5 wt% YAG	1900 °C	3.072	8.11	93.14	6H-SiC	YAG
	1950 °C	3.223	9.34	98.53	6H-SiC	YAG
	2000 °C	3.201	11.23	96.55	6H-SiC	YAG
SiC/10 wt% YAG	1900 °C	3.131	8.93	94.85	6H-SiC	YAG
	1950 °C	3.216	10.66	98.41	6H-SiC	YAG
	2000 °C	3.203	13.07	96.22	6H-SiC	YAG

**Table 2:** Mechanical properties of SiC/YAG composite ceramics.

Sample	Sintering Conditions	Bending Strength (MPa)	Fracture Toughness (MPa·m^1/2^)	Reference
SiC/5 wt% YAG	1900 °C/30 MPa/1 h/Ar	383 ± 38	4.51	This work
	1950 °C/30 MPa/1 h/Ar	675 ± 43	5.56	
	2000 °C/30 MPa/1 h/Ar	451 ± 49	4.80	
SiC/10 wt% YAG	1900 °C/30 MPa/1 h/Ar	379 ± 41	4.45	
	1950 °C/30 MPa/1 h/Ar	649 ± 45	5.52	
	2000 °C/30 MPa/1 h/Ar	493 ± 37	4.79	
SiC/10 wt% YAG	1950 °C/1 h/Ar	437	4.9	[40]
SiC/5 wt% TiN	1950 °C/1 h/Ar	545.2	\	[41]

## Data Availability

The data presented in this study are available on request from the corresponding author (due to privacy).
